# Potential methylation-regulated genes and pathways in hepatocellular neoplasm, not otherwise specified

**DOI:** 10.3389/fonc.2022.952325

**Published:** 2022-09-21

**Authors:** Shengmei Zhou, Meng Li, Dejerianne Ostrow, David Ruble, Leo Mascarenhas, Bruce Pawel, Jonathan David Buckley, Timothy J. Triche

**Affiliations:** ^1^ Department of Pathology and Laboratory Medicine, Children’s Hospital Los Angeles, Los Angeles, CA, United States; ^2^ Keck School of Medicine, University of Southern California, Los Angeles, CA, United States; ^3^ USC Libraries Bioinformatics Services, University of Southern California, Los Angeles, CA, United States; ^4^ Cancer and Blood Disease Institute, Division of Hematology/Oncology, Department of Pediatrics, Children’s Hospital Los Angeles, Los Angeles, CA, United States

**Keywords:** hepatocellular neoplasm, not otherwise specified, RNAseq, genome-wide DNA methylation, EPIC850, hepatoblastoma, hepatocellular carcinoma

## Abstract

**Background and Aims:**

The molecular basis of hepatocellular neoplasm, not otherwise specified (HCN-NOS) is unknown. We aimed to identify gene expression patterns, potential methylation-regulated genes and pathways that characterize the tumor, and its possible relationship to hepatoblastoma and hepatocellular carcinoma (HCC).

**Approach & Results:**

Parallel genome-wide profiling of gene expression (RNAseq) and DNA methylation (EPIC850) was performed on 4 pairs of pre-treatment HCN-NOS tumors and adjacent non-tumor controls. 2530 significantly differentially expressed genes (DEGs) were identified between tumors and controls. Many of these DEGs were associated with hepatoblastoma and/or HCC. Analysis Match in Ingenuity Pathway Analysis determined that the gene expression profile of HCN-NOS was unique but significantly similar to that of both hepatoblastoma and HCC. A total of 27,195 CpG sites (CpGs) were significantly differentially methylated (DM) between tumors and controls, with a global hypomethylation pattern and predominant CpG island hypermethylation in promotor regions. Aberrant DNA methylation predominated in Developmental Process and Molecular Function Regulator pathways. Embryonic stem cell pathways were significantly enriched. In total, 1055 aberrantly methylated (at CpGs) and differentially expressed genes were identified, including 25 upstream regulators and sixty-one potential CpG island methylation-regulated genes. Eight methylation-regulated genes (*TCF3*, *MYBL2*, *SRC*, *HMGA2*, *PPARGC1A*, *SLC22A1*, *COL2A1* and *MYCN*) had highly consistent gene expression patterns and prognostic value in patients with HCC, based on comparison to publicly available datasets.

**Conclusions:**

HCN-NOS has a unique, stem-cell like gene expression and DNA methylation profile related to both hepatoblastoma and HCC but distinct therefrom. Further, 8 methylation-regulated genes associated with prognosis in HCC were identified.

## Introduction

Hepatocellular neoplasm, not otherwise specified (HCN-NOS), is a provisional diagnostic entity that describes a subset of highly malignant pediatric liver tumors (1). HCN-NOS typically develops in patients aged 4-15 years and demonstrates heterogeneous histologic features neither typical for hepatoblastoma nor for hepatocellular carcinoma (HCC) (1, 2). The entity was originally named as transitional liver cell tumor (TLCT), as it was believed to be a neoplastic continuum from hepatoblastoma to HCC (3). A genomic study of three such tumors suggested that HCN-NOS might be a genetically derailed progeny of hepatoblastoma (4). Our prior clinicopathological study of 11 patients with HCN-NOS suggested that HCN-NOS might be a subtype of hepatoblastoma with focal HCC-like histology and a high-risk clinical profile (2). An accurate diagnosis of HCN-NOS is critical for selection of appropriate treatment regimens. However, it often poses a diagnostic challenge even to experienced pediatric pathologists owing to a lack of specific molecular markers to distinguish it from either hepatoblastoma or HCC. New diagnostic and prognostic markers, and novel breakthrough therapies directed against specific molecular alterations and or tumorigenic pathways of HCN-NOS may improve diagnosis and treatment.

Previous studies demonstrated that somatic gene mutations are exceedingly rare in pediatric liver cancer (4, 5). Aberrant DNA methylation, one of the most common molecular alterations in human cancer, contributes to the onset and progression of many pediatric cancers (6–8). Aberrant DNA methylation usually occurs early in tumorigenesis (9), is tumor specific, is relatively stable in fixed samples over time (10), and may classify cancers, as well as subtypes of a specific cancer (8). As such, DNA methylation studies hold great promise for the development of diagnostic, prognostic, and therapeutic biomarkers.

In this study, we examined the global patterns of gene expression and DNA methylation changes in HCN-NOS tumors, performed functional analysis of altered genes with a particular focus on methylation-regulated genes, and cross-referenced selected gene sets against comparable publicly available gene expression data from hepatoblastoma and HCC.

## Materials and methods

This study was approved by the Children’s Hospital Los Angeles institutional review board (CHLA-17-00158).

### Tumor samples

Four pre-treatment HCN-NOS tumors (3 frozen samples and one formalin-fixed, paraffin-embedded (FFPE) sample) and corresponding surrounding non-tumor liver tissue (all FFPE) were profiled in this study. All tumor samples were clinically and pathologically characterized, and then macro-dissected to enrich for neoplastic cellularity.

### DNA and RNA extraction

For frozen samples, DNA was extracted using Gentra Puregene Tissue Kit (Qiagen Inc., Valencia, CA) and RNA was extracted with Qiagen RNeasy Mini RNA Extraction Kit (Qiagen, Hilden, Germany). For FFPE samples, DNA was isolated using the QIAamp DNA FFPE Tissue Kit (QIAGEN) and RNA was extracted with the Beckman Coulter FormaPure RNA Extraction Kit (Beckman Coulter, Brea, CA). Both DNA and RNA were subjected to standard quality control procedures to confirm that the samples were adequate for DNA and RNA sequencing.

### RNAseq sequencing and analysis

Libraries were prepared using the KAPA RNA Hyper Prep Kit (KAPA Biosystems, Wilmington, MA) with an initial input quantity of 100 ng purified RNA. Hybridization was performed using the Twist Biosciences Human Core Exome and the Human RefSeq Panel probes and the Fast Hybridization and Wash Kit (Twist Biosciences, San Francisco, CA). Final libraries were quantified with the Agilent High Sensitivity D1000 ScreenTape Assay (Agilent, Santa Clara, CA) and sequenced (paired end, 2x100 bp) on the Illumina NextSeq 500 (Illumina, San Diego, CA) with 100 million reads per sample.

RNAseq data were analyzed using Partek Flow, version 10 (Partek Inc., Missouri, USA). Raw sequencing reads were first trimmed for base quality using the Quality Score method (base positions with Phred scores less than 20 were trimmed from both ends; trimmed reads shorter than 25 nt were excluded from downstream analyses). Trimmed reads were aligned to human genome GRCh38 using STAR 2.6.1d with Gencode 32 as guidance. Aligned reads were then quantified to Gencode 32 using Partek E/M. The control sample from case 3 was removed for further analysis due to poor quality. Genes with fewer than 10 raw read counts in all 7 remaining samples were removed from further analysis. Raw reads were normalized using Upper Quartile normalization (11) with an offset of 1. Differential expression was assessed by Partek’s Gene Specific Analysis (GSA), which applies voom weighting of normalized counts to account for variations in precision (12), followed by linear modeling using limma (13). The differentially expressed genes (DEGs) were selected with cutoffs of false discovery rate (FDR) < 0.05 and fold change (FC) of |FC| >3.

### DNA methylation profiling and analysis

DNA bisulfite conversion, post-bisulfite quality control (QC) testing, Illumina Restoration Kit, and array hybridization to the Illumina Infinium MethylationEPIC BeadChip array (EPIC850) for data production were performed according to the manufacturer instructions at USC Norris Molecular Genomic Core. 500 ng FFPE-DNAs or 1µg frozen DNAs in 45 µl volume from each sample were submitted.

Methylation data were analyzed using Partek^®^ Genomics Suite^®^ software (PGS), v7.18 (Partek Inc. Missouri, USA). Probes located at a documented single nucleotide polymorphism site or determined to be cross-reactive with other probes were removed from the analysis (14). Probes with a detection p-value > 0.05, based on comparison of the observed β to background variation, were also eliminated and the β values for the remaining 792017 probes were converted to M-values (M-value = log2(β/(1 − β)). Differential methylation (tumor vs. normal) was assessed using 2-way ANOVA of tumor/normal and patient ID. Significantly differentially methylated (DM) probes were defined as those with p < 0.01 (approximating an FDR < 0.2) and absolute M-value FC > 1.5.

### Functional analysis

To investigate biologically significant processes, fold-change values and p-value of the sets of differentially expressed genes from RNAseq profiling and differentially methylated genes from DNA methylation profiling were uploaded to the Ingenuity Pathway Analysis (IPA; v.1.13, Qiagen, Inc.) package separately and both cross-referenced against the global gene network in the Ingenuity Knowledge Base. For each biological function and/or disease in the database, the proportion of function/disease-associated genes that were differentially expressed was compared to the differentially expressed rates for all other genes using Fischer’s exact test to provide a p-value for association between the function/disease and differential expression. An associated z score indicted the direction of the activation of a canonical pathway or functions and regarded as significant if its absolute value was equal or greater than 2.

### Validation of gene expression in independent public HCC datasets

We identified genes of high interest, as those showing significant differential expression, differential methylation, or both, in HCN-NOS. Expression patterns and prognostic associations of selected genes of interest were evaluated in public HCC datasets (LIHC) compiled from The Cancer Genome Atlas (TCGA) along with Therapeutically Applicable Research to Generate Effective Treatments (TARGET) and the Genotype-Tissue Expression (GTEx) databases, using UCSC Xena (http://xena.ucsc.edu/). No comparable datasets of hepatoblastoma are available from these sources.

### Validation of the expression of 4 up-regulated genes by immunohistochemical staining

To validate some gene expression data from RNAseq at protein levels, we performed IHC staining of glypican 3, spalt-like transcription factor 4 (SALL4), high mobility group AT-hook 2 (HMGA2) and Forkhead box M1 (FoxM1) in the 4 paired tumors and controls (see Supporting data for details). The gene expression of these 4 proteins were all up-regulated in our study and appropriate antibodies to them were available to us.

## Results

### Clinicopathological features of patients

The detailed clinical information of the 4 patients (case 1-4) included in the current study and their tumor characteristics, along with histological features (pre- and post-treatment), have been previously reported (corresponding to case no. 6, 7, 10 and 11, respectively) (2). Briefly, they were all male with the age ranging 4–11 years (median age: 7 years). All patients had multiple tumors and high alpha-fetal protein (AFP) (269,000 to 1,280,000 ng/dl). More representative histological pictures from these tumors are shown on **Supporting Figure S1**.

### Characterization of gene expression profiling of HCN-NOS

#### Many aberrantly expressed genes were associated with hepatoblastoma and or HCC

**Figure 1:**
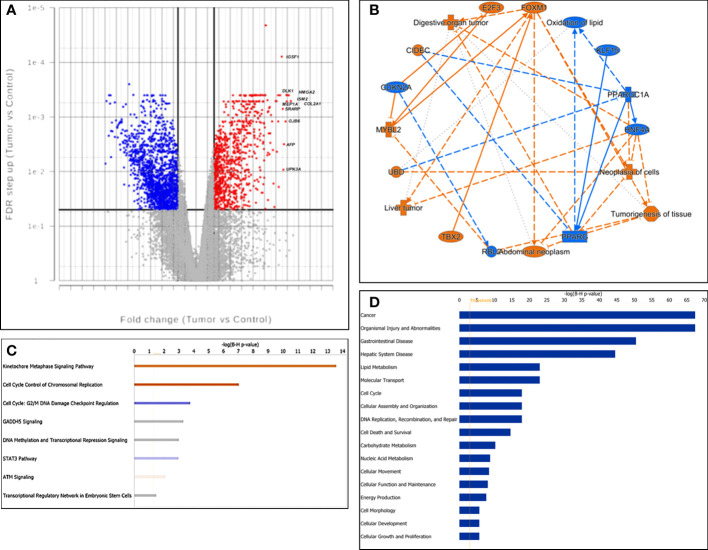
Characterization of gene expression profiling. **(A)** Volcano plot of gene expression. X-axis: fold change difference; y-axis: FDR (false discovery rate); vertical lines: fold change >|3|; horizontal line: the significance cutoff (FDR p-value = 0.05). The 10 most upregulated genes were labeled. **(B)** The graphic summary, a network of interconnected biological findings by IPA analysis. Predicted activation was labeled in orange, while predicted inhibition was labeled in blue. **(C)** Canonical signaling pathways enriched by differentially expressed genes. Z-scores were presented by colors. Red: activation; blue: suppression; gray: unable to make a confident prediction; and white: unable to make a prediction. **(D)** The barchart displayed the most significantly enriched diseases and functions across the dataset, listed from the most significant to the least.

We identified 2530 DEGs between tumors and controls (**Supporting Table S1**), 895 being significantly up-regulated and 1635 down-regulated (**Figure 1A**). The 10 most up-regulated genes (based on FC) were *COL2A1*, *DLK1*, *MEP1A*, *ISM2*, *HMGA2*, *GJB6*, *AFP, UPK3A*, *SRARP* and *IGSF1*, and the 10 most down-regulated genes were *UGT2B17*, *CNDP1*, *SAA2-SAA4*, *HAMP*, *SLC22A1*, *HPGD*, *SERTM2*, *CLEC4M*, *CYP1A2* and *CLEC4G*.

Cross-referencing the significantly up-regulated gene set against the 20 most up-regulated hepatoblastoma genes reported by Sumazin et al (5), we found 14 matches: *DKK1*, *GPC3*, *HMGA2*, *REG3A*, *DLK1*, *COL2A1*, *DKK4*, *TNFRSF19*, *NPNT*, *CDCA7*, *SLC7A11*, *SERPINI1*, *AFP* and *LIN28B*. A similar comparison with a 16 gene hepatoblastoma classifier reported by Cairo et al (15), yielded 12 genes with concordant expression patterns as in the C2 group (more aggressive one): *GHR*, *APCS*, *ALDH2*, *C1S*, *CYP2E1*, *APOC4*, *HPD*, *RPL10A*, *BUB1*, *IGSF1*, *AFP* and *DUSP9*.

Other significantly upregulated genes such as *SALL4, FOXM1*, *MYCN*, *MEP1A* and *MYBL2* have also been reported to be significantly elevated in HCC and associated with tumor progression (16–20). Interestingly, while beta-catenin and TERT protein expression levels were previously observed to be increased in HCN-NOS (2), gene expression changes in both *CTNNB1* (FDR = 0.24, FC = 1.57) and *TERT* (FDR = 0.09, FC = 26.51) didn’t reach the threshold for selection in our dataset, likely due to a combination of the high threshold imposed and the small number of samples in this study.

#### Enriched canonical pathways, disease and functions

We next performed functional analysis of the DEGs by IPA. The graphic summary, a network composed of the interconnected biological findings by IPA analysis, is shown in **Figure 1B**. It was predicted that the genes such as *FOXM1* and *MYBL2* were activated while *HNF4A* and *PPARGC1A* were inhibited in tumor cells, which drove liver tumor, digestive organ tumor, abdominal neoplasm, tumorigenesis of tissue, neoplasia of cells and oxidation of lipid.

Canonical Pathways Analysis revealed that Kinetochore Metaphase Signaling Pathway (Category: Cellular Growth, Proliferation and Development) and Cell Cycle Control of Chromosomal Replication (Category: Cell Cycle Regulation) were predicted to be significantly activated while Cell cycle: G2/M DNA Damage Checkpoint Regulation (Category: Cell Cycle Regulation) and STAT3 Pathway were predicted to be inhibited. Both DNA methylation and Transcriptional Regression Signaling and Transcriptional Regulatory Network in Embryonic Stem Cells were significantly enriched with uncertain prediction (**Figure 1C**).

The bar chart in **Figure 1D** shows that the top enriched disease was “Cancer” and the top enriched functions included “Lipid Metabolism”, “Molecular Transport”, and “Cell cycle” in our dataset.

#### DEGs as upstream regulators

To explore what drove the gene expression changes, we analyzed upstream regulators of the DEGs by IPA. A total of 209 potential upstream regulators were identified with a B-H corrected P-value < 0.01 (**Supporting Table S2**). Among them, tumor oncogenes such as *FOXM1* (Z score = 4.8, FC = 14.3), *MYBL2* (Z score =2.7, FC = 34.1) and *E2F1* (Z score = 4.6, FC =21.9) were significantly upregulated and predicted to be activated, while the tumor suppressor gene *PPARGC1A* (Z score = -3.0, FC = -45.3) was downregulated and predicted to be prominently inhibited. Additional non-significantly differentially expressed tumor oncogenes such as *MYC* and *YAP1* (21) were also predicted to be activated, while tumor suppressor genes such as *RB1* and *Let-7*, were predicted to be inhibited in HCN-NOS (**Supporting Figure S2A**).

#### The gene expression profiling of HCN-NOS was unique but significantly similar to that of both hepatoblastoma and HCC

We performed IPA’s Analysis Match to cross-reference the HCN-NOS gene expression dataset against over 96,000 publicly available gene expression datasets (such as GEO and Human disease) to identify those with significantly similar or dissimilar gene expression profiles. The reference datasets with the strongest similarity to our dataset were those related to HCC. Filtering the reference datasets to include only those based on liver disease/normal comparisons, 17 reference datasets with normalized Z scores greater than 30 were identified, including 11 HCC datasets (with GSE124535, an RNAseq comparison of paired tumor/non-tumor samples from 35 adults with HCC, being most significant, Z = 61.2), 1 hepatoblastoma, 3 liver cancer NOS, and 2 acute liver failure datasets. The single significantly dissimilar dataset (GSE61276, a genomewide expression study of 92 adult and 14 fetal liver samples) had a Z score of -30.58 (**Supporting Figure S3A**).

By further comparing Canonical Pathways (**Supporting Figure S3B**), Upstream Regulators (**Supporting Figure S3C**), Causal Networks, and Diseases and Functions between our dataset and the reference datasets, we found that there were some important similarities and differences among HCC, HCN-NOS and hepatoblastoma. Together these findings suggest that HCN-NOS have a unique gene expression profile thought it has substantial similarities to both hepatoblastoma and HCC.

### Characterization of DNA methylation profiling of HCN-NOS

#### DNA methylation exhibited a global hypomethylation, with predominate CpG island hypermethylation in promotor regions

**Figure 2:**
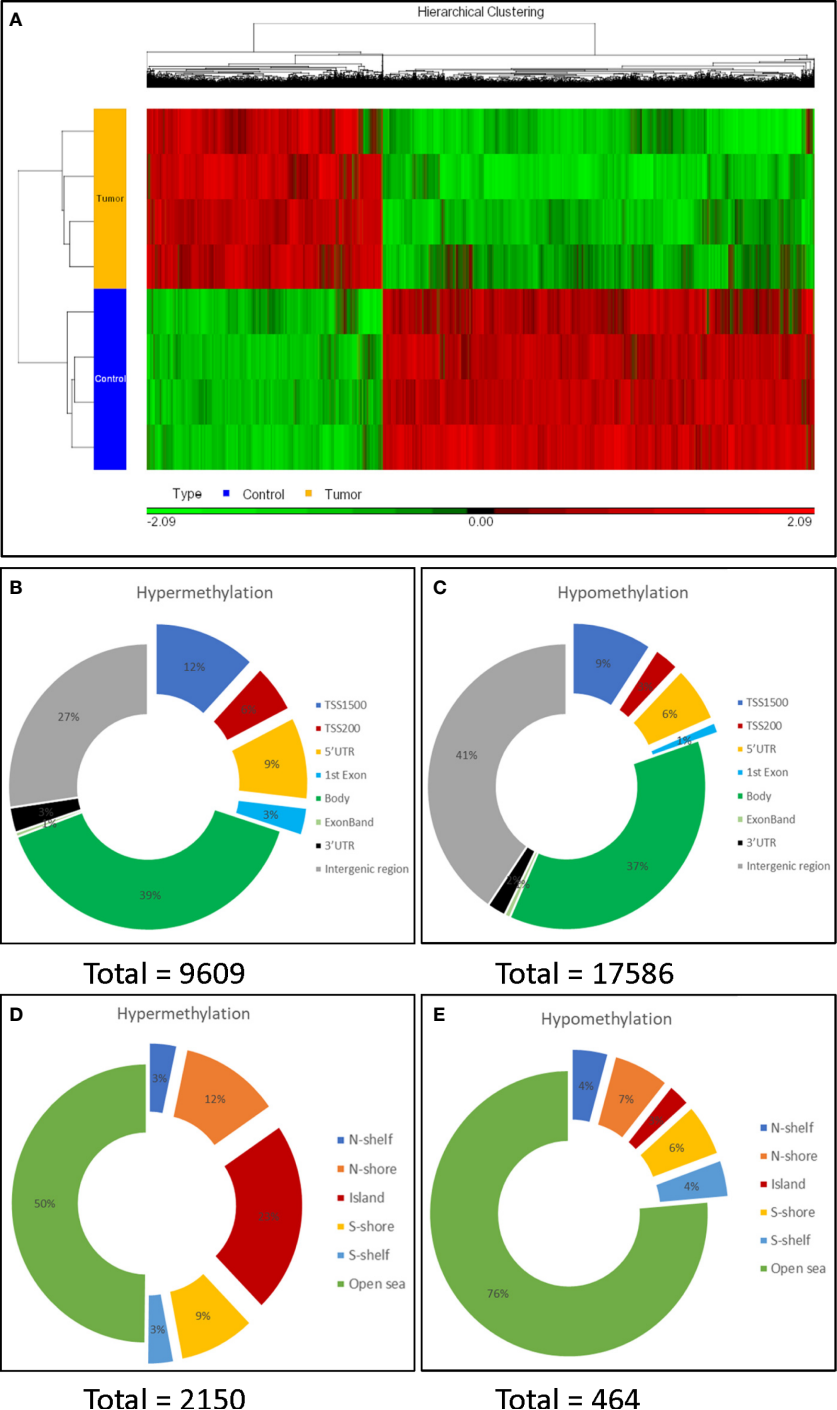
Characterization of DNA methylation profiling. **(A)** Heat map of unsupervised hierarchical clustering of the 27,195 differentially methylated CpG sites. There were more hypo- (green) than hyper-methylated CpG sites (red) in tumors compared to the controls. **(B, C)**: Distribution of the hypermethylated and hypomethylated CpG sites over genomic elements. **(D, E)**: Distribution around CpG islands. The CpG island shores (flanking 2k bases) and shelves (flanking 2-4k base), divided into N (north, or 5’) and S (3’); more distant istermed ‘open sea’. Promotor regions were preferentially hypermethylated and half of hypermethylated CpG sites were within (23%) or adjacentto a CpG island (a shelf or shore), while 76% of hypomethylated CpG sites occurred in the open sea.

DNA methylation profiling identified 27,195 significantly differentially methylated CpG sites (CpGs) (3.43% of the total 792,017 CpGs, mapping to 8,423 genes; **Supporting Table S3**). Of these, 9,609 CpGs (35.3%) were hypermethylated and 17,586 (64.7%) CpGs were hypomethylated in HCN-NOS tumors, consistent with a global hypomethylation pattern (**Figure 2A**).

Mapping differentially methylated (DM) CpGs to genomic elements, 30% of hypermethylated CpGs, but only 19% of hypomethylated CpGs, were found within promoter regions (defined as TSS1500, TSS200, 5’-UTR and 1st exon) (**Figures 2B**, **C**), indicating that the promotor region was preferentially hypermethylated. In contrast, the preferential hypomethylation sites were gene bodies and intergenic regions.

More hypermethylated CpGs (n = 2,150) than hypomethylated CpGs (n = 464) were found in, or adjacent to, CpG islands. Moreover, half of the hypermethylated CpGs were within (23%) or flanking CpG islands. In contrast, only 3% of the hypomethylated CpGs were within a CpG island, while most (76%) were found in open sea regions (**Figures 2D**, **E**).

CpG islands are clusters of CpGs, largely unmethylated throughout the genome in normal cells. Most CpG islands span sites of transcription initiation and have a strong association with regulation of gene activity (22, 23). We also performed CpG island analysis by first summarizing all the probes belonging to a CpG island to get the mean methylation level and then used ANOVA to compare the tumors and controls. With cutoffs of p<0.01 and M-value >1.5, we found that there were 864 significantly DM CpG islands (in proximity to 712 genes), with more hypermethylated (n=646) than hypomethylated (n=218) CpG islands, consistent with the findings from individual CpG analysis.

#### Aberrant DNA methylation patterns predominated in developmental process and molecular function regulator pathways.

We next performed gene ontology (GO) enrichment analysis of the 8,423 genes with significantly DM CpGs using PGS. All GO terms with > 5 genes were analyzed. We found that “developmental process” was the most enriched biological process (**Supporting Figure S4A**). As for molecular function, “transporter activity” and “molecular function regulators” were the top enriched (**Supporting Figure S4B**). GO enrichment analysis of the 712 genes with DM CpG islands revealed that “developmental process” was also the top enriched biological process (**Supporting Figure S4C**). Moreover, “molecular function regulators” was the predominantly affected molecular function (**Supporting Figure S4D**). Further analysis of “molecular function regulators” showed that the transcription regulators were predominately hypermethylated (a detailed Forest plot is shown in **Supporting Figure S4E**). The results suggest that aberrant DNA methylation may be a common and key event underlying the tumorigenesis of HCN-NOS through affecting developmental process and molecular function regulators, especially hypermethylation of transcription regulators.

#### Embryonic stem cell pathways were significantly enriched

**Figure 3:**
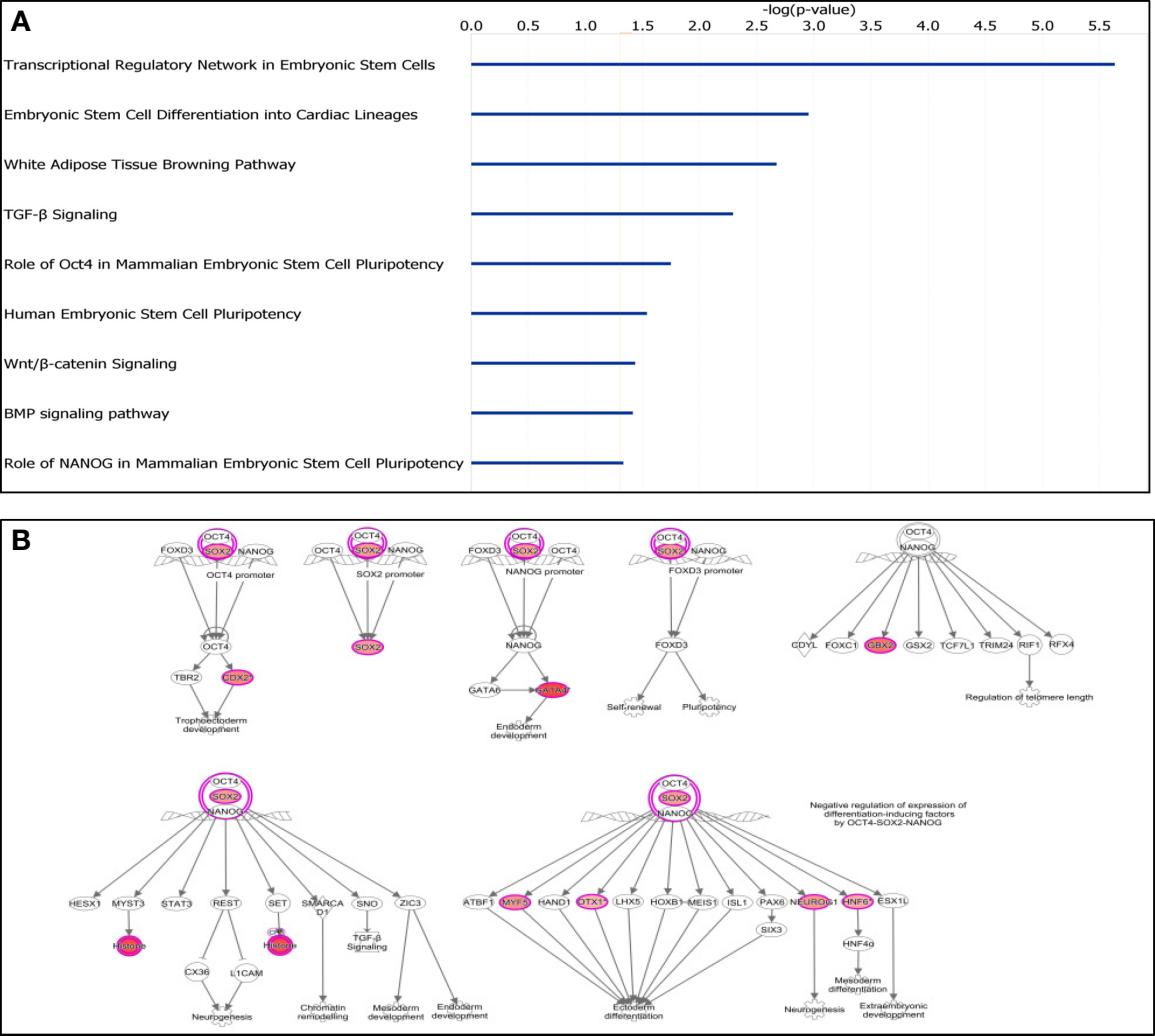
Characterization of differentially methylated CpG islands. **(A)** Top significantly affected canonical pathways. Transcriptional Regulatory Network in Embryonic Stem Cells was the highest ranking enriched canonical pathway. **(B)** IPA analysis of transcriptional regulatory network in embryonal stem cells. Nine stem cell related genes were all hypermethylated (in red).

Given the direct role of CpG island methylation on gene expressions, we used IPA to further study the 712 genes with adjacent DM CpG islands. Interestingly, the most enriched canonical pathway for this gene set was “Transcriptional Regulatory Network in Embryonic Stem Cells” (P = 2.32×10^−6^) (**Figure 3A**). Moreover, “Role of Oct4 in Mammalian Embryonic Stem Cell Pluripotency”, “Human Embryonic Stem Cell Pluripotency”, “Wnt/β-catenin Signaling” and “Role of NANOG in Mammalian Embryonic Stem Cell Pluripotency” were all significantly enriched. Furthermore, 9 stem cell related genes (*SOX2, CDX2, GATA4, GBX2, H4C6, MYF5, NEUROG1, ONECUT1* and *OTX1*) showed significant CpG island hypermethylation in tumors (**Figure 3B**). Interestingly, none of these 9 genes showed significant gene expression changes.

### Characterization of aberrantly methylated-differentially expressed genes

**Figure 4:**
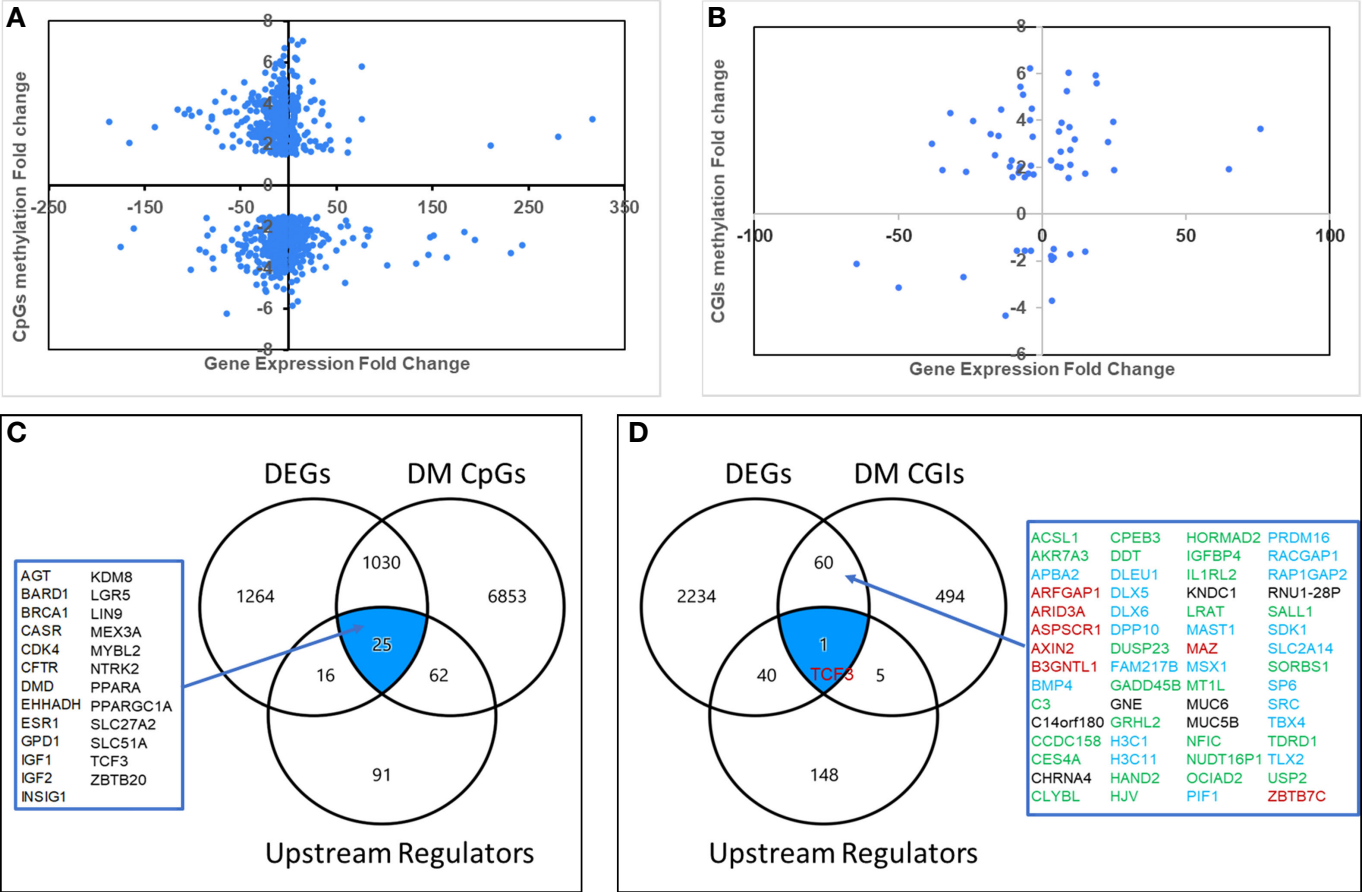
Aberrantly methylated-differentially expressed genes. Quadrant scattered plots showed differentially expressed genes (DEGs) with corresponding differentially methylated (DM) CpG sites (CpGs) **(A)** and DM CpG islands (CGIs) **(B)**. Venn diagrams showed 25 genes **(C)** identified by integrating DEGs, DM CpGs and Upstream Regulators, 61 genes **(D)** by overlapping DEGs and DM CpG islands, and one gene (TCF3) by overlapping DEGs, DM CpG islands and Upstream Regulators. Green: hypermethylated/downregulated genes, Blue: hypermethylated/upregulated genes, Red: hypomethylated/upregulated genes, Black: hypomethylated/downregulated genes.

To further explore the potential biological relevance of DM CpGs, we integrated the datasets of DM CpGs and DEGs to identified 1,055 genes that were both differentially methylated and differentially expressed. Of these, 608 (57.6%) showed an inverse relationship between methylation and expression, 395 being hypermethylated and down-regulated, and 213 hypomethylated and up-regulated. The remaining 447 genes showed a positive relationship between CpG methylation and gene expression levels, including 105 hypermethylated and up-regulated, and 342 hypomethylated and down-regulated (**Figure 4A**; **Supporting Table S4**).

Since upstream regulators are critical for gene expression, we hypothesized that they could be potential biomarkers. To explore potential DNA methylation-regulated upstream regulators, we overlapped the datasets of DM CpGs, DEGs and the 209 upstream regulators. A total of 25 differentially methylated, differentially expressed upstream regulators were identified (**Figure 4C**), and 15 of them (60%) had an inverse relationship between CpG methylation and gene expression levels.

To further explore potential CpG island methylation regulated genes, we integrated the datasets of DM CpG islands and DEGs. As a result, 61 genes were identified (**Figures 4B, D**), including 25 hypermethylated and downregulated, 21 hypermethylated and upregulated, 8 hypomethylated and upregulated, and 7 hypomethylated and downregulated. An inverse relationship between aberrant DNA methylation in CpG islands and gene expression levels was present in 54% genes.

The DNA methylation status and location of the 61 potential CpG island methylation-regulated genes were further visualized using the UCSC Genome Browser. We found that the DNA methylation status of many genes was in accordance with the findings observed in HepG2 (a human liver cancer cell line). Representative examples of 4 genes (*GADD45B*, *DLX6*, *AXIN2* and *GADD45B*) are shown in **Supporting Figure S5**. Interestingly, both *GADD45B* and *DLX6* had hypermethylated CpG islands within promoter regions, but *GADD45B* had decreased gene expression while *DLX6* had increased gene expression. Notably, reduced expression of *GADD45B* (a tumor suppressor gene) due to promoter methylation was also observed in HCC (24). Hypomethylated CpG islands of *AXIN2* were also within promoter regions and associated with increased gene expression. *OCIAD2* (a tumor suppressor gene) showed a hypermethylated and down-regulated expression. Reduced expression of *OCIAD2* by DNA hypermethylation was reported to play an important role in HCC tumor growth and invasion (25).

Overlapping DEGs, DM CpG islands and the upstream regulators yielded a single common gene: *TCF3* (**Figure 4D**), a ubiquitous transcription regulator, which was associated with many DEGs through interaction with genes such as *MYC*, *LEF1*, *FOXO1* and *CDKN2A.* The mechanistic network of *TCF3* is shown in **Supporting Figure S2B**.

### Expression patterns of selected DEGs in independent public HCC datasets

Using UCSC Xena, we first analyzed four above-described potentially important upstream regulators, *FOXM1, MYBL2, E2F1* and *PPARGC1A.* We found that *FOXM1*, *MYBL2* and *E2F1* were all significantly upregulated while *PPARGC1A* was significantly downregulated in HCC, fully consistent with the results observed in HCN-NOS (**Supporting Figure 6A**).

We next investigated 9 genes (*SLC22A1*, *CYP1A2, AFP*, *DLK1*, *COL2A1, HMGA2, MYCN, CLEC4M* and *CLEC4G*), which were among the most up-regulated or down-regulated genes with aberrant methylation. All genes except DLK1 showed significant differences between tumors and controls in LIHC datasets, matching the results observed in HCN-NOS (**Supporting Figure 6B**). We further explored the gene expression of the 61 potential CpG island methylation-regulated genes and found that that expression of *HAND2*, *CES4A*, *SORBS1*, *GADD45B* and *C14orf180* were all significantly down-regulated, while *SP6, SRC*, *MAZ*, *TCF3* were all significantly up-regulated in HCCs compared to normal liver controls, in complete agreement of the results observed in HCN-NOS (**Supporting Figure 6C**).

**Figure 5:**
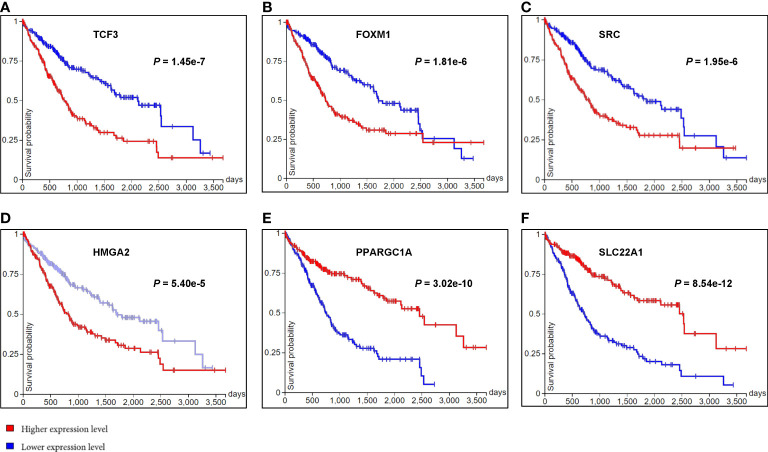
Kaplan-Meier plots of the overall survival of HCC patients between higher and lower gene expression levels of six differentially expressed genes observed in HCN-NOS. The horizontal axis represented the overall survival time in days and the vertical axis represented overall survival probability. Higher gene expression levels of *TCF3* **(A)**, *FOXM1* **(B)**, *SRC* **(C)** and *HMGA2* **(D)** were all associated with lower overall survival while higher gene expression levels of *PPARGC1A* **(E)** and *SLC22A1* **(F)** were associated with higher overall survival of HCC patients.

Subsequently, the overall survival of HCC patients between higher and lower gene expression levels of the above mentioned 21 genes were analyzed in public HCC datasets from TCGA using Kaplan-Meier (Log-rank test) *via* UCSC Xena. We found the gene expression levels of 10 of them were significantly associated with the overall survival of HCC patients. They were 8 potential DNA methylation regulated genes (*TCF3*, *MYBL2*, *SRC*, *HMGA2*, *PPARGC1A*, *SLC22A1*, *COL2A*1 and *MYCN*) and 2 non-DNA methylation related important genes (*FOXM1* and *E2F1*) (**Figures 5A–F** and **Supporting Figure S7**). For example, the gene expression of *TCF3* (**Figure 5A**), a gene significantly hypomethylated and upregulated in HCN-NOS, had a significant negative correlation with OS in patients with HCC, meaning patients with higher gene expression of *TCF3* were associated with lower overall survival. Meanwhile, *PPARGC1A* (**Figure 5E**), a significantly hypermethylated and downregulated gene and an upstream regulator in HCN-NOS, had a significant positive correlation with OS, meaning patients with higher gene expression of *PPARGC1A* were associated with higher overall survival in HCC. The findings suggest that these 10 genes may be candidates as HCN-NOS prognostic markers.

### Four up-regulated genes showed protein overexpression

**Figure 6:**
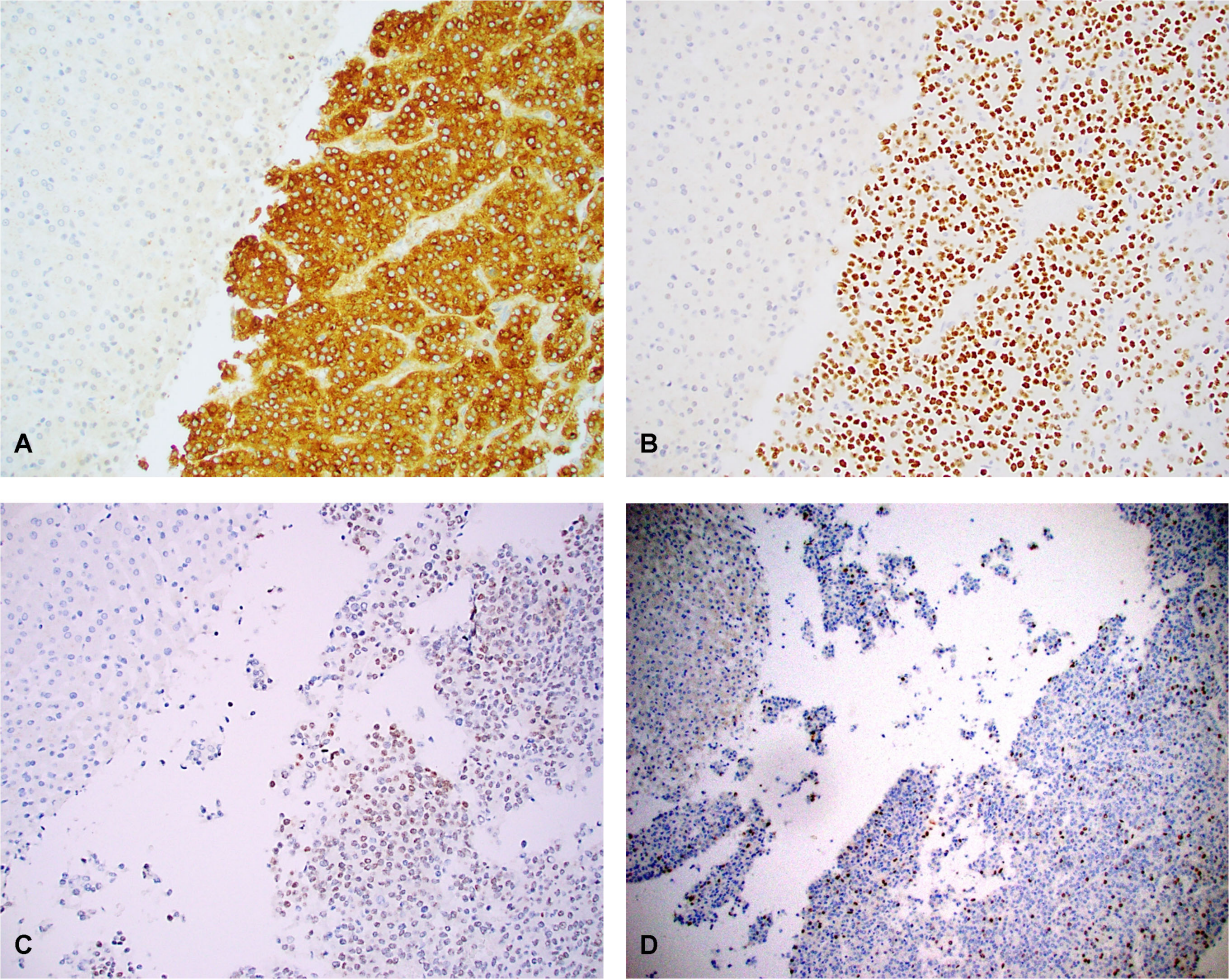
Overexpression of glypican 3 **(A)**, SALL4 **(B)**, HMGA2 **(C)** and FOXM1 **(D)** in case 3 with both uninvolved normal liver (left upper) and adjacent HCN-NOS tumor (right side). Tumor cells were positive for glypican 3 (moderate to strong cytoplasmic staining), SALL4 (moderate to strong nuclear staining), HMGA2 (patchy, weak to strong nuclear staining) and FOXM1 (scattered strong nuclear staining), In contrast, the normal liver cells (left upper) showed no staining for glypican 3, SALL4, HMGA2 and FOXM1. The results were consistent with the gene expression data obtained by RNAseq. Original magnification: 100 x for all.

We found all 4 HCN-NOS tumors were uniformly positive for glypican 3 (encoded by *GPC3*) (moderate to strong cytoplasmic staining) (**Figure 6A**), SALL4 (moderate to strong nuclear staining) (**Figure 6B**), HMGA2 (weak to strong nuclear staining) (**Figure 6C**) and FOXM1 (scattered strong nuclear staining, ranging from 5-30%) (**Figure 6D**) by IHC. In contrast, all matched normal liver controls were completely negative for glypican 3, SALL4, HMGA2 and FOXM1 by IHC analysis. The results were consistent with the gene expression data obtained by RNAseq.

## Discussion

This is the first study to characterize genome-wide gene expression and DNA methylation profiles in paired normal liver and HCN-NOS tumor tissue, to our knowledge. Studying both methylome and transcriptome using a paired study method allowed us to analyze the biological relevance of DNA methylation changes. We found that HCN-NOS has a unique stem-cell like gene expression and DNA methylation profile related to both hepatoblastoma and HCC but distinct therefrom. And many potential important DNA methylation-regulated genes and pathways were identified.

It is still uncertain if HCN-NOS is a distinct entity or a subtype of hepatoblastoma or HCC. The gene expression pattern by RNAseq in HCN-NOS has not been explored previously. In this study, we identified 2530 DEGs, and there were more down-regulated genes than up-regulated ones (1636 vs. 895). Many aberrantly expressed genes were associated with hepatoblastoma and or HCC. When compared to the hepatoblastoma 16 gene signature (15), the overall expression pattern of HCN-NOS was compatible well with the more aggressive hepatoblastoma. Canonical pathways in the categories of “Cellular Growth”, “Cell Cycle Regulation”, and “Proliferation and Development”, which are essential to tumorigenesis, were predominantly enriched. Remarkably, many tumor oncogenes such as *MYC* and *YAP1 (* 21) were predicted to be activated upstream regulators while many tumor suppressor genes such as *RB1* and *Let-7* were predicted to be inhibited upstream regulators. By Analysis Match, we further found that the gene expression profiling of HCN-NOS was unique but significantly similar to that of both hepatoblastoma and HCC, and more closely matched to that of HCC than that of hepatoblastoma. Recently, Sumazin et al. (26) also found that HCN-NOS has combined molecular features of hepatoblastoma and HCC. These findings are in concordance with the overlapping histological features of HCN-NOS between hepatoblastoma and HCC (2), and support the assumption that HCN-NOS may be a transitional tumor from hepatoblastoma to HCC (3).

HCN-NOS had a global hypomethylation pattern, in line with both HCC (27) and heptatoblastoma (28, 29). Its preferential hypomethylation sites were gene bodies and intergenic regions. A global hypomethylation may lead to carcinogenesis by increasing chromosomal instability (30) as well as by aberrant activation of oncogenes (31). We also observed that CpG islands in the promoter region were predominately hypermethylated in HCN-NOS. Hypermethylation of CpG islands located in the promoter and first exon regions of tumor suppressor genes has been established as one of the most common mechanisms for gene regulation in cancers (23, 32).

It has been suggested that stem cells may be the precursors from which cancer cells are derived (33) and genes involved in regulation of a stem cell state may be more vulnerable to aberrant DNA methylation (34). The histologic features of HCN-NOS may resemble various stages of liver development, suggestive of arising from embryonic cells (1, 2). Interestingly, we found that the developmental process and molecular function regulators were predominantly affected by aberrant DNA methylation. What’s more, multiple canonical pathways related to regulation of embryonic stem cell were significantly enriched. And nine stem cell transcription factors including *SOX2* were significantly hypermethylated but none of them showing significant gene expression changes. Our data imply that aberrant DNA methylation of genes regulating embryonic stem cell-like cells may play critical roles in tumorigenesis of HCN-NOS in a somewhat complex, counterintuitive manner.

Most previous methylation studies of hepatoblastoma studied only one or a few genes at a time by methylation-specific PCR. As a result, aberrant DNA methylation at the *APC, CDH1, MT1G, RASSF1A* and *SOCS1* promoters were reported in hepatoblastoma (35, 36). Limited genome-wide methylation analysis by HM450 found that differentially methylated genes were involved with liver cell differentiation and cancer (29), and four tumor suppressor genes (*GPR180*, *MST1R*, *OCIAD2*, and *PARP6*) were potentially related to progression in hepatoblastomas (37). However, no consistent results had been observed across studies and none of the above studies included a parallel gene expression study. As for HCC, there are several DNA methylation studies on adult type with wide variations in methylation patterns, while DNA methylation study on pediatric HCC is lacking. In this study, we used EPIC850, a highly reliable genomic platform with more comprehensive DNA methylation analysis capacity than HM450 (38). We analyzed altered methylation not only at CpG sites but also within CpG islands. Furthermore, we focused on aberrantly methylated-differentially expressed genes instead of the top-ranked genes based on the absolute value of different methylation.

A total of 1055 aberrantly methylated (at CpGs) - differentially expressed genes were identified, including 25 upstream regulators and 61 potential CpG island methylation-regulated genes. Surprisingly, only OCIAD2 (hypermethylated and down-regulated) of the above noted 9 suppressor genes in hepatoblastoma was among the 1055 genes. Using public HCC datasets, we found that 21 potential important DEGs had consistent expression patterns in HCC. Eight potential DNA methylation regulated genes had highly consistent gene expression patterns and prognostic values in HCC patients. Due to paired samples and stringent cut-off values, theoretically, many of these genes might be potential diagnostic/prognostic biomarkers. However, further investigation in larger numbers of samples and functional studies of these genes is required for development of a potential clinical diagnositic or prognostic biomarker profile.

The main limitations of the study include the relatively small number of cases, their retrospective nature and inconsistency of tissue preservation. Despite these limitations, unsupervised hierarchical clustering analysis of both DM CpGs and DEGs showed a distinct separation between HCN-NOS and controls. The consistent expression patterns of at least 21 genes were observed in public HCC datasets, thus validating our overall approach. And 4 gene expression data from RNAseq were validated at protein levels by immunostaining.

Our study provides novel insights into the molecular basis of HCN-NOS. Aberrant DNA methylation may play a critical role in the tumorigenesis of HCN-NOS. Many DNA methylation-regulated genes identified in this study could serve as the basis for continuing research on novel diagnostic, prognostic and therapeutic biomarkers for HCN-NOS.

## Data availability statement

The datasets presented in this study can be found in online repositories. The data presented in the study are deposited in the the National Center for Biotechnology Information Gene Expression Omnibus (GEO). RNAseq and DNA methylation data are accessible through the GEO Series accession number GSE195664 and GSE199747, respectively.

## Ethics statement

The studies involving human participants were reviewed and approved by the Children’s Hospital Los Angeles institutional review board. Written informed consent from the participants’ legal guardian/next of kin was not required to participate in this study in accordance with the national legislation and the institutional requirements.

## Author contributions

SZ conceived and implemented the study, analyzed data, and wrote the manuscript. ML analyzed the data, helped with some figures and revised the manuscript. DO and DR processed specimens and collected data. LM and BP provided patient care and clinical data. JB double checked data and revised the manuscript. TT made substantial contributions to conception and design and revised the manuscript. All authors contributed to the article and approved the submitted version.

## Funding

This work was supported in part by grants UL1TR001855 from the National Center for Advancing Translational Science (NCATS) of the U.S. National Institutes of Health (SC CTSI Voucher grants 8092-RGP010905 & 8092-RGP011497). The content is solely the responsibility of the authors and does not necessarily represent the official views of the National Institutes of Health.

## Conflict of interest

The authors declare that the research was conducted in the absence of any commercial or financial relationships that could be construed as a potential conflict of interest.

## Publisher’s note

All claims expressed in this article are solely those of the authors and do not necessarily represent those of their affiliated organizations, or those of the publisher, the editors and the reviewers. Any product that may be evaluated in this article, or claim that may be made by its manufacturer, is not guaranteed or endorsed by the publisher.

## Supplementary material

Supplementary Figure S1 Representative histological features of the 4 cases. **(A)** Tumor from case 1 was composed of large polygonal cells with round to oval hyperchromatic vesicular nuclei, small nucleoli, moderate amounts of eosinophilic cytoplasm, numerous mitoses and arranged mainly in acini and trabeculae. **(B)** Resection tumor from case 2 revealed sheets of medium sized polygonal cells with round hyperchromatic vesicular nuclei, inconspicuous nucleoli, focal cytoplasmic vacuolation and frequent mitoses. **(C)** Tumor section from case 3 demonstrated normal liver parenchyma (left upper) and adjacent tumor (right side), which was composed of small to medium sized atypical cells with vesicular nuclei, inconspicuous nucleoli, moderate amounts of clear to eosinophilic cytoplasm and increased mitoses. **(D)** Tumor from case 4 showed sheets of medium to large polygonal cells with large nuclei, prominent nucleoli and increased mitotic activity. **(A–D)** Hematoxylin-eosin stain; Original magnification: 200 x for all.

Supplementary Figure S2 **(A)** Network derived from analysis of the upstream regulator YAP1 of RNAseq data. Note that CTNNB1, YAP1 and MYC were predicted to be activated while RB1 and Let-7, were predicted to be inhibited. Five genes from the network was overlaid with Wnt/β-catenin Signaling. **(B)** The mechanistic network of TCF3. The predicted effects of TCF3 and its interaction with other upstream regulators (middle) were displayed. The related gene expression changes observed in our dataset were shown in the bottom of the hierarchy. Up-regulated genes were labeled in red, while down-regulated genes were labeled in green. The saturation of color was correlated with the fold changes of the gene (high saturation means high fold change and low saturation means low fold change). Ellipses: transcription regulators, rhombuses: enzymes, trapezoids: transporters, double circles: a complex/group, and circles: others.

Supplementary Figure S3 Analysis Match results. A total of 18 public comparisons from 15 datasets showed significant similar or dissimilar to our dataset, and 14 of 18 from HCC, based on the absolute normalized Z score >30 **(A)**. Each of the first four colored columns represents similarity score of each type of signature to the dataset of HCN-NOS. The fuchsia color indicates similarity and cyan color indicates dissimilarity. The first scoring column (“CP”) is for the Canonical Pathway signature, the second (“UR”) is for Upstream Regulators, the third (“CN”) is for Causal Networks, and the last (“DE”) is for Downstream Effects (i.e., Diseases and Functions). The final of the fuchsia and blue column is the average of those four signature matches. The white and purple columns to the right of the Z-score columns display the right-tailed Fisher's Exact Test p-value for each of the signature matches. Heatmaps of Comparing Canonical Pathways **(B)** and Upstream Regulators **(C)** of our dataset (2530 GSA) vs. 18 public comparisons.

Supplementary Figure S4 Gene Ontology (GO) enrichment analysis of biological processes and molecular function of the 27,195 DM CpGs **(A, B)** and the 864 DM CpG islands **(C, D)**, respectively. Different GO-terms are shown in vertical axis with Enrichment score in parenthesis. Developmental process and molecular function regulators were predominantly affected by aberrant DNA methylation. **(E)** Forest plot of GO enrichment analysis specially on the molecular function regulators based on DM CpG islands. Red: Hypermethylation, Green: Hypomethylation. The transcription regulators were predominately hypermethylated.

Supplementary Figure S5 UCSC Genome Browser view of DNA methylation and added corresponding RNA fold change (FC) of 4 genes (GADD45B **(A)**, DLX6 **(B)**, AXIN2 **(C)**, and OCIAD2 **(D)**. From the top down: scale bar, size of region in kilobases of DNA; chromosome number and exact coordinates from the hg19 genome build; CpG Islands; our DNA methylation data (FC); gene track; DNA methylation data on HepG2 (a human liver cancer cell line) using Reduced Representation Bisulfite Sequencing (RRBS) from ENCODE project (red = 100% of molecules sequenced are methylated, yellow = 50% of molecules sequenced are methylated, green = 0% of molecular sequenced are methylated); chromatin state segmentations on HepG2 cell line, using a consensus merge of the segmentations produced by the ChromHMM and Segway software (bright red = predicted promoter region including TSS, light red = predicted promoter flanking region, orange = predicted enhancer, yellow = predicted weak enhancer or open chromatin cis regulatory element, blue = CTCF enriched element, dark green = predicted transcribed region, gray = predicted repressed or low activity region). Hypermethylated CpG islands of GADD45B and DLX6 were both within promoter regions (bright red on the track of H2pG2 combined), but had opposite effects on RNA expression. Hypomethylated CpG islands of AXIN2 were also within promoter regions with increased RNA expression. And OCIAD2 showed a hypermethylated (uncertain chromatin state) and down-regulated pattern.

Supplementary Figure S6 Expression patterns of potential important genes in HCC datasets. **(A)** Image analysis and box plots of gene expression of four differentially expressed upstream regulators in HCC datasets. FOXM1, MYBL2 and E2F1 were all significantly up-regulated while PPARGC1A was significantly down-regulated in HCC. **(B)** Box plots of 9 genes (aberrantly methylated and profoundly differentially expressed) in HCC. All genes except DLK1 showed significant differences between HCC and normal liver controls. **(C)** Box plots of 9 potential DNA CpG island methylation-regulated genes in HCC. The gene expression of HAND2, CES4A, SORBS1, GADD45B and C14orf180 were all significantly downregulated while SP6, SRC, MAZ and TCF3 were all significantly upregulated in HCC tumors compared to the normal liver controls.

Supplementary Figure S7 Kaplan-Meier plots of the overall survival of HCC patients between higher and lower gene expression levels of 4 differentially expressed genes identified in HCN-NOS. The horizontal axis represents the overall survival time in days and the vertical axis represents overall survival probability. Higher gene expression levels of COL2A1 **(A)**, MYCN **(B)**, MYBL2 **(C)** and E2F1 **(D)** were all associated with lower overall survival.
