# Supplementary figures and images for: Potential methylation-regulated genes and pathways in hepatocellular neoplasm, not otherwise specified
Source: Front Oncol. 2022 Sep 21;12:952325. doi: 10.3389/fonc.2022.952325 (PMC9532972; doi:10.3389/fonc.2022.952325)

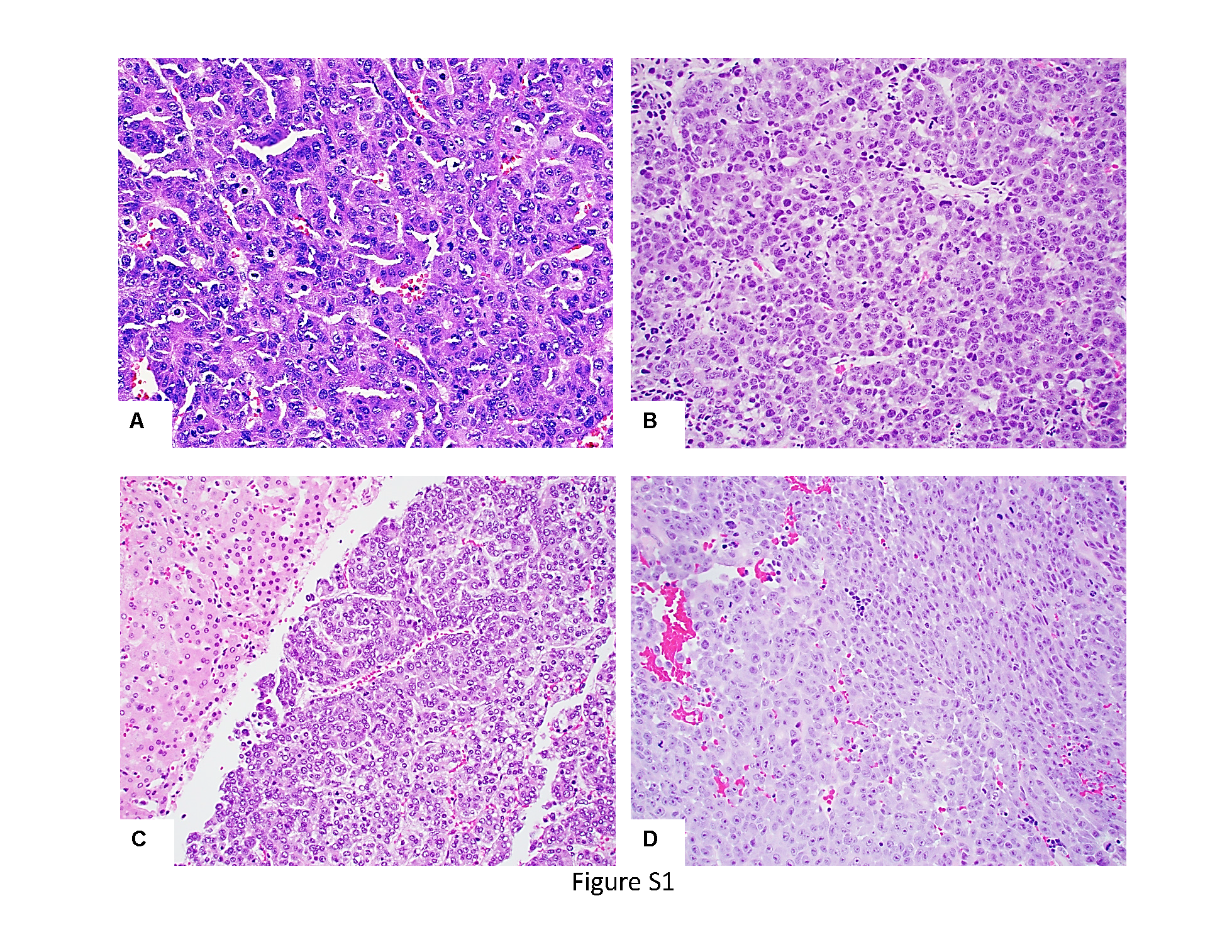

Supplement: Supplementary Figure S1 — Representative histological features of the 4 cases. (A) Tumor from case 1 was composed of large polygonal cells with round to oval hyperchromatic vesicular nuclei, small nucleoli, moderate amounts of eosinophilic cytoplasm, numerous mitoses and arranged mainly in acini and trabeculae. (B) Resection tumor from case 2 revealed sheets of medium sized polygonal cells with round hyperchromatic vesicular nuclei, inconspicuous nucleoli, focal cytoplasmic vacuolation and frequent mitoses. (C) Tumor section from case 3 demonstrated normal liver parenchyma (left upper) and adjacent tumor (right side), which was composed of small to medium sized atypical cells with vesicular nuclei, inconspicuous nucleoli, moderate amounts of clear to eosinophilic cytoplasm and increased mitoses. (D) Tumor from case 4 showed sheets of medium to large polygonal cells with large nuclei, prominent nucleoli and increased mitotic activity. (A–D) Hematoxylin-eosin stain; Original magnification: 200 x for all. [file DataSheet_1.zip › Image 1.TIFF]

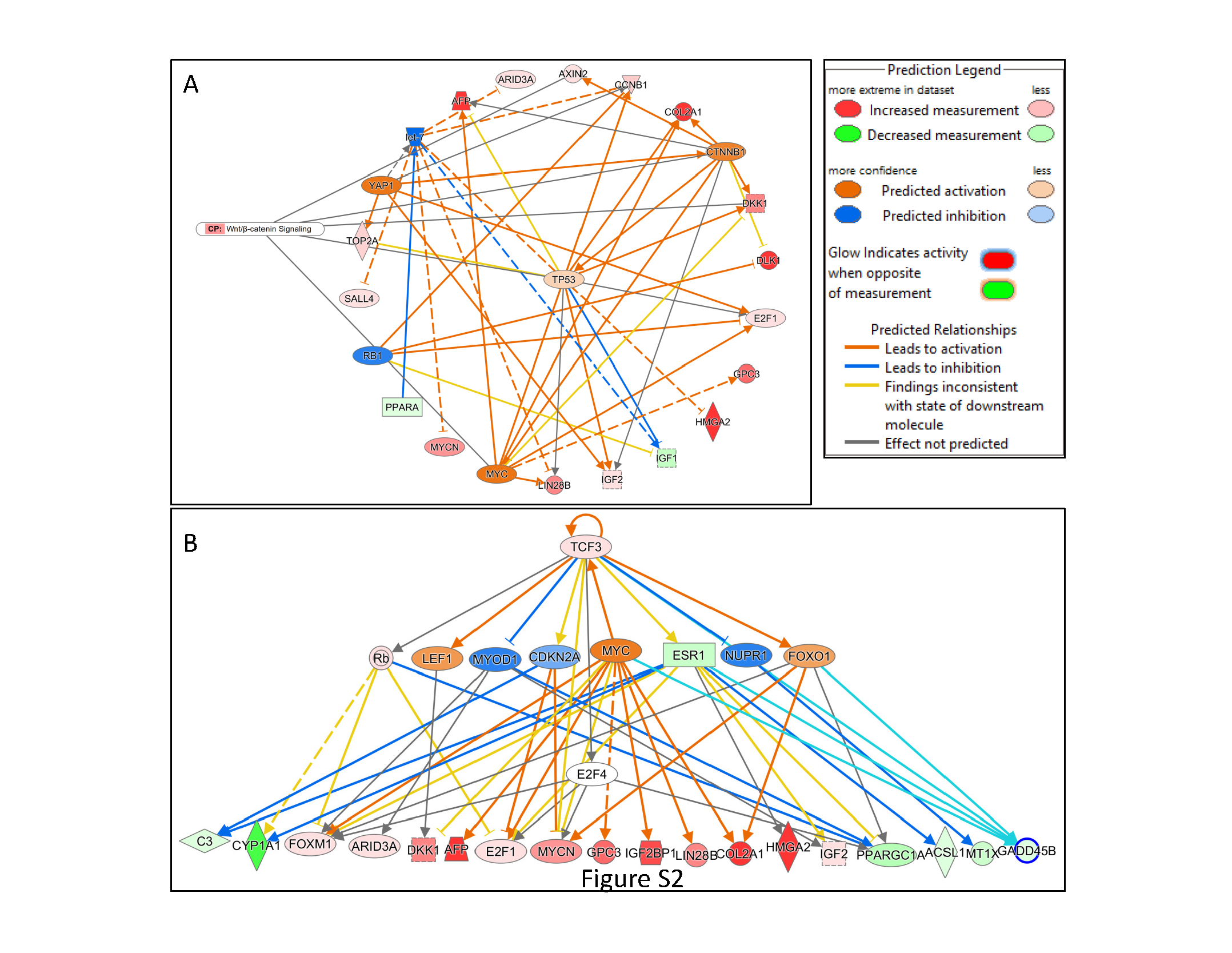

Supplement: Supplementary Figure S1 — Representative histological features of the 4 cases. (A) Tumor from case 1 was composed of large polygonal cells with round to oval hyperchromatic vesicular nuclei, small nucleoli, moderate amounts of eosinophilic cytoplasm, numerous mitoses and arranged mainly in acini and trabeculae. (B) Resection tumor from case 2 revealed sheets of medium sized polygonal cells with round hyperchromatic vesicular nuclei, inconspicuous nucleoli, focal cytoplasmic vacuolation and frequent mitoses. (C) Tumor section from case 3 demonstrated normal liver parenchyma (left upper) and adjacent tumor (right side), which was composed of small to medium sized atypical cells with vesicular nuclei, inconspicuous nucleoli, moderate amounts of clear to eosinophilic cytoplasm and increased mitoses. (D) Tumor from case 4 showed sheets of medium to large polygonal cells with large nuclei, prominent nucleoli and increased mitotic activity. (A–D) Hematoxylin-eosin stain; Original magnification: 200 x for all. [file DataSheet_1.zip › Image 2.TIF]

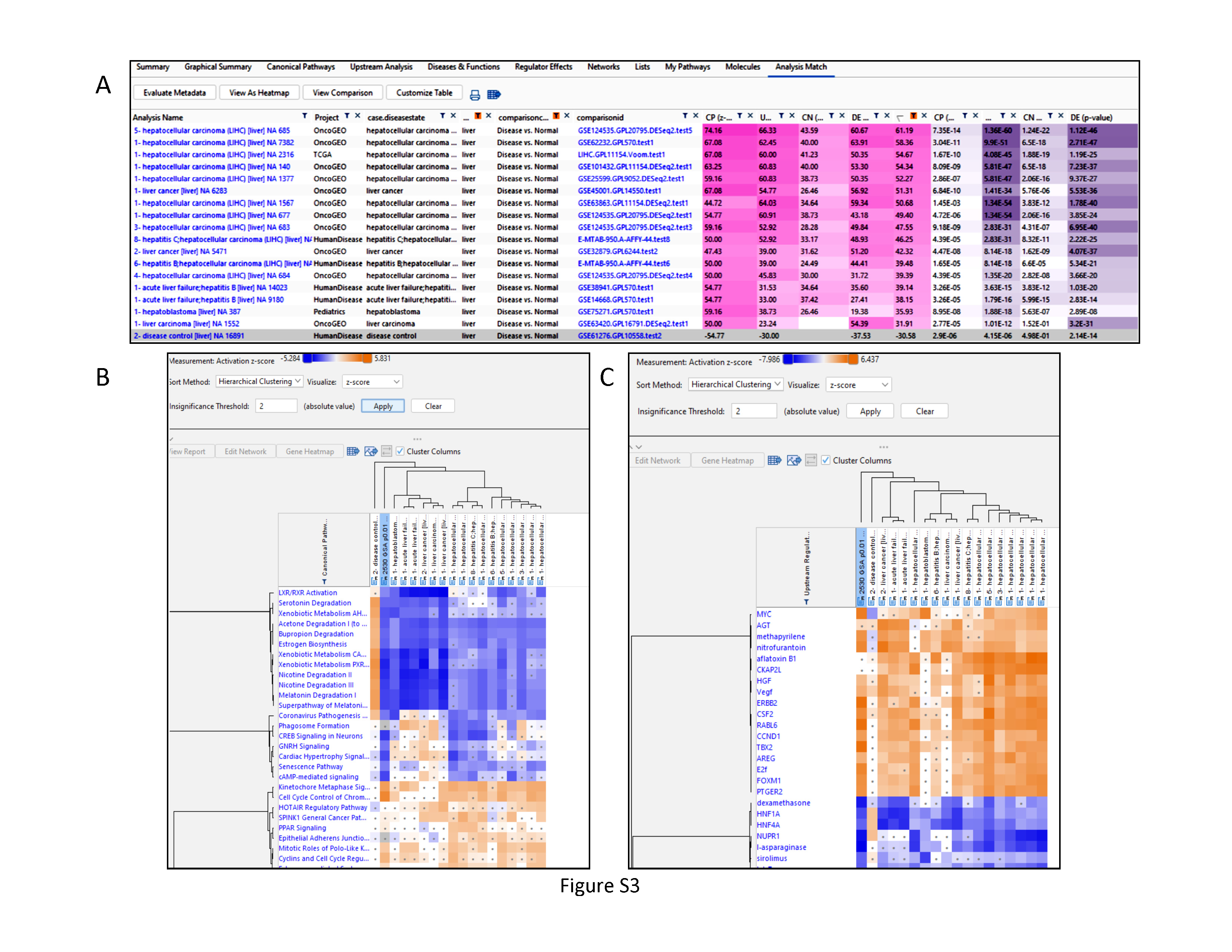

Supplement: Supplementary Figure S1 — Representative histological features of the 4 cases. (A) Tumor from case 1 was composed of large polygonal cells with round to oval hyperchromatic vesicular nuclei, small nucleoli, moderate amounts of eosinophilic cytoplasm, numerous mitoses and arranged mainly in acini and trabeculae. (B) Resection tumor from case 2 revealed sheets of medium sized polygonal cells with round hyperchromatic vesicular nuclei, inconspicuous nucleoli, focal cytoplasmic vacuolation and frequent mitoses. (C) Tumor section from case 3 demonstrated normal liver parenchyma (left upper) and adjacent tumor (right side), which was composed of small to medium sized atypical cells with vesicular nuclei, inconspicuous nucleoli, moderate amounts of clear to eosinophilic cytoplasm and increased mitoses. (D) Tumor from case 4 showed sheets of medium to large polygonal cells with large nuclei, prominent nucleoli and increased mitotic activity. (A–D) Hematoxylin-eosin stain; Original magnification: 200 x for all. [file DataSheet_1.zip › Image 3.TIF]

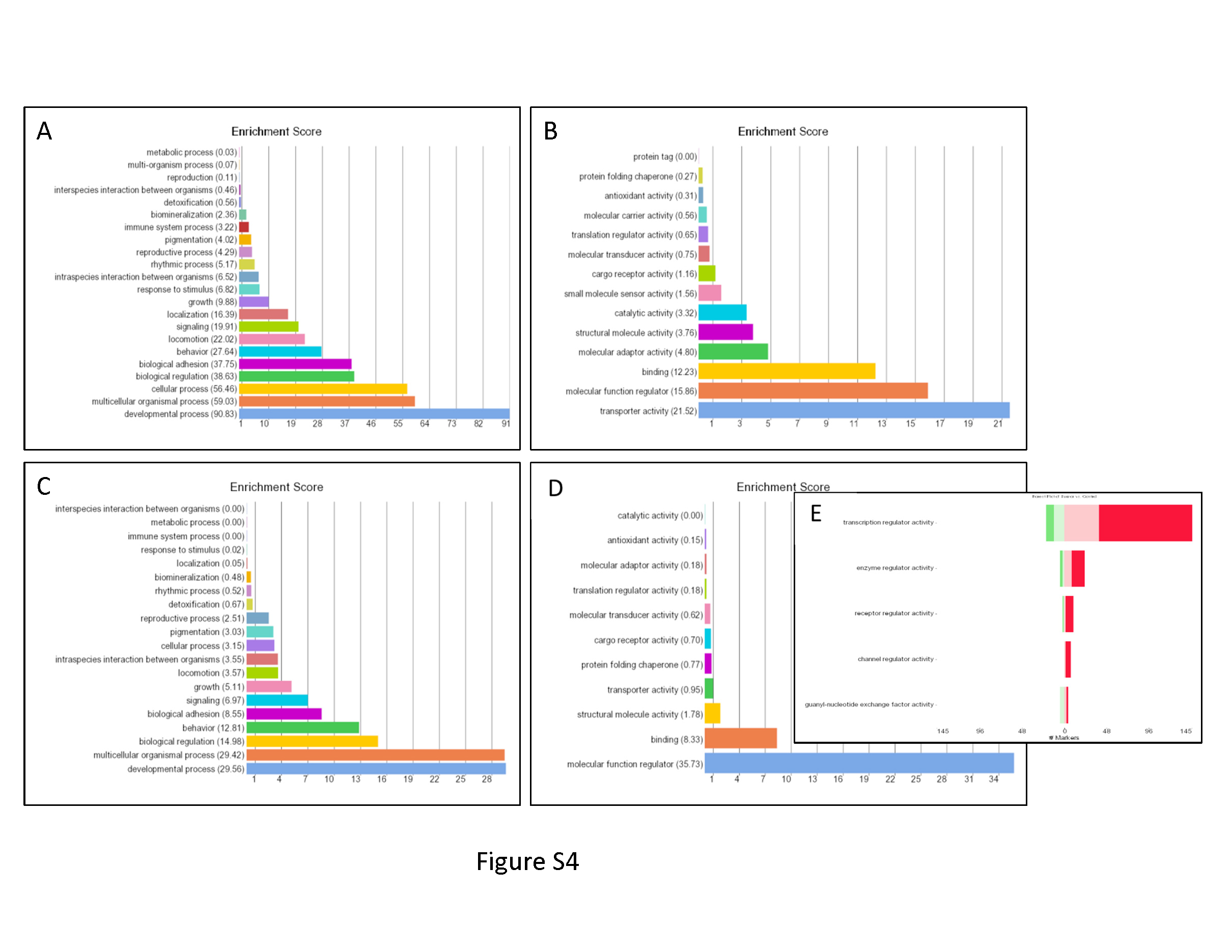

Supplement: Supplementary Figure S1 — Representative histological features of the 4 cases. (A) Tumor from case 1 was composed of large polygonal cells with round to oval hyperchromatic vesicular nuclei, small nucleoli, moderate amounts of eosinophilic cytoplasm, numerous mitoses and arranged mainly in acini and trabeculae. (B) Resection tumor from case 2 revealed sheets of medium sized polygonal cells with round hyperchromatic vesicular nuclei, inconspicuous nucleoli, focal cytoplasmic vacuolation and frequent mitoses. (C) Tumor section from case 3 demonstrated normal liver parenchyma (left upper) and adjacent tumor (right side), which was composed of small to medium sized atypical cells with vesicular nuclei, inconspicuous nucleoli, moderate amounts of clear to eosinophilic cytoplasm and increased mitoses. (D) Tumor from case 4 showed sheets of medium to large polygonal cells with large nuclei, prominent nucleoli and increased mitotic activity. (A–D) Hematoxylin-eosin stain; Original magnification: 200 x for all. [file DataSheet_1.zip › Image 4.TIF]

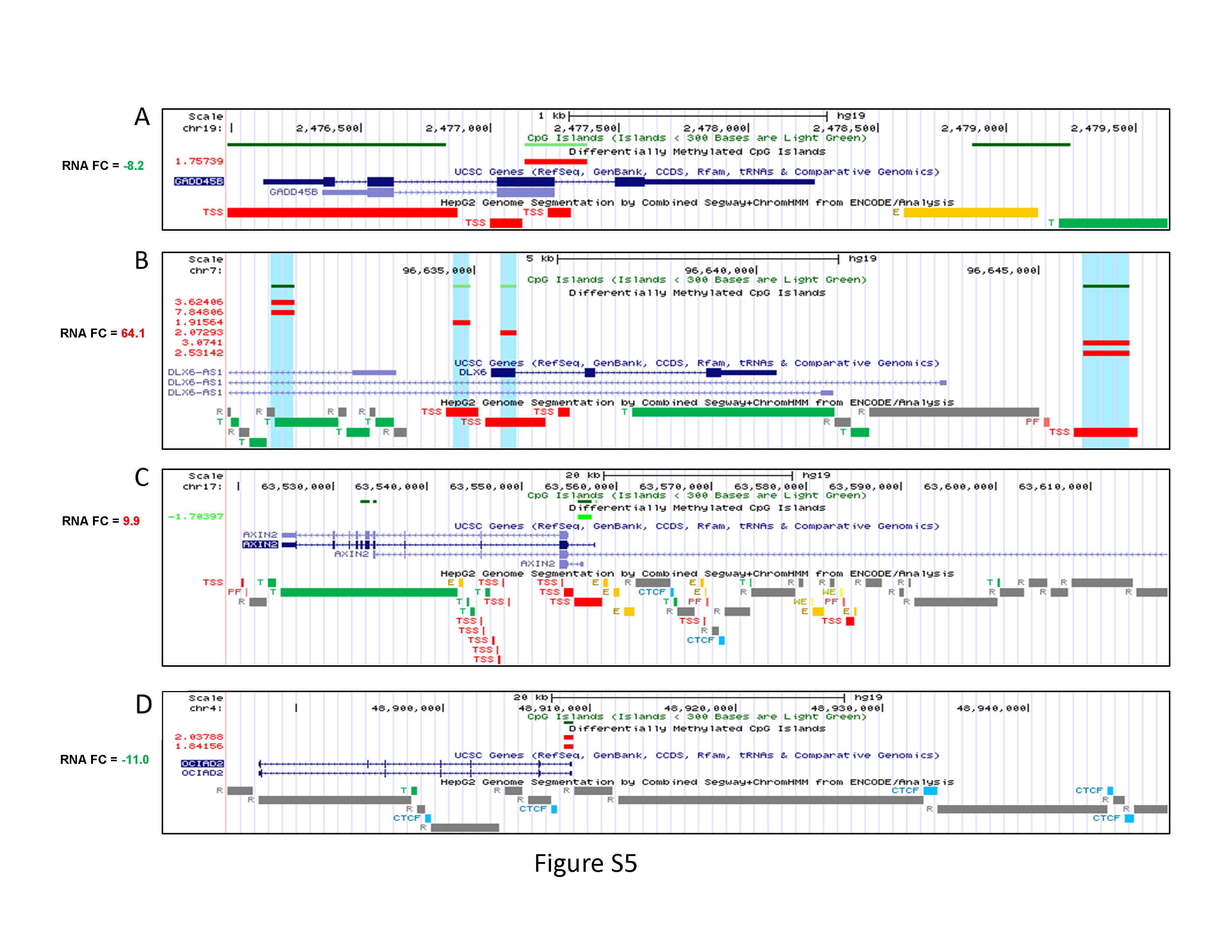

Supplement: Supplementary Figure S1 — Representative histological features of the 4 cases. (A) Tumor from case 1 was composed of large polygonal cells with round to oval hyperchromatic vesicular nuclei, small nucleoli, moderate amounts of eosinophilic cytoplasm, numerous mitoses and arranged mainly in acini and trabeculae. (B) Resection tumor from case 2 revealed sheets of medium sized polygonal cells with round hyperchromatic vesicular nuclei, inconspicuous nucleoli, focal cytoplasmic vacuolation and frequent mitoses. (C) Tumor section from case 3 demonstrated normal liver parenchyma (left upper) and adjacent tumor (right side), which was composed of small to medium sized atypical cells with vesicular nuclei, inconspicuous nucleoli, moderate amounts of clear to eosinophilic cytoplasm and increased mitoses. (D) Tumor from case 4 showed sheets of medium to large polygonal cells with large nuclei, prominent nucleoli and increased mitotic activity. (A–D) Hematoxylin-eosin stain; Original magnification: 200 x for all. [file DataSheet_1.zip › Image 5.TIF]

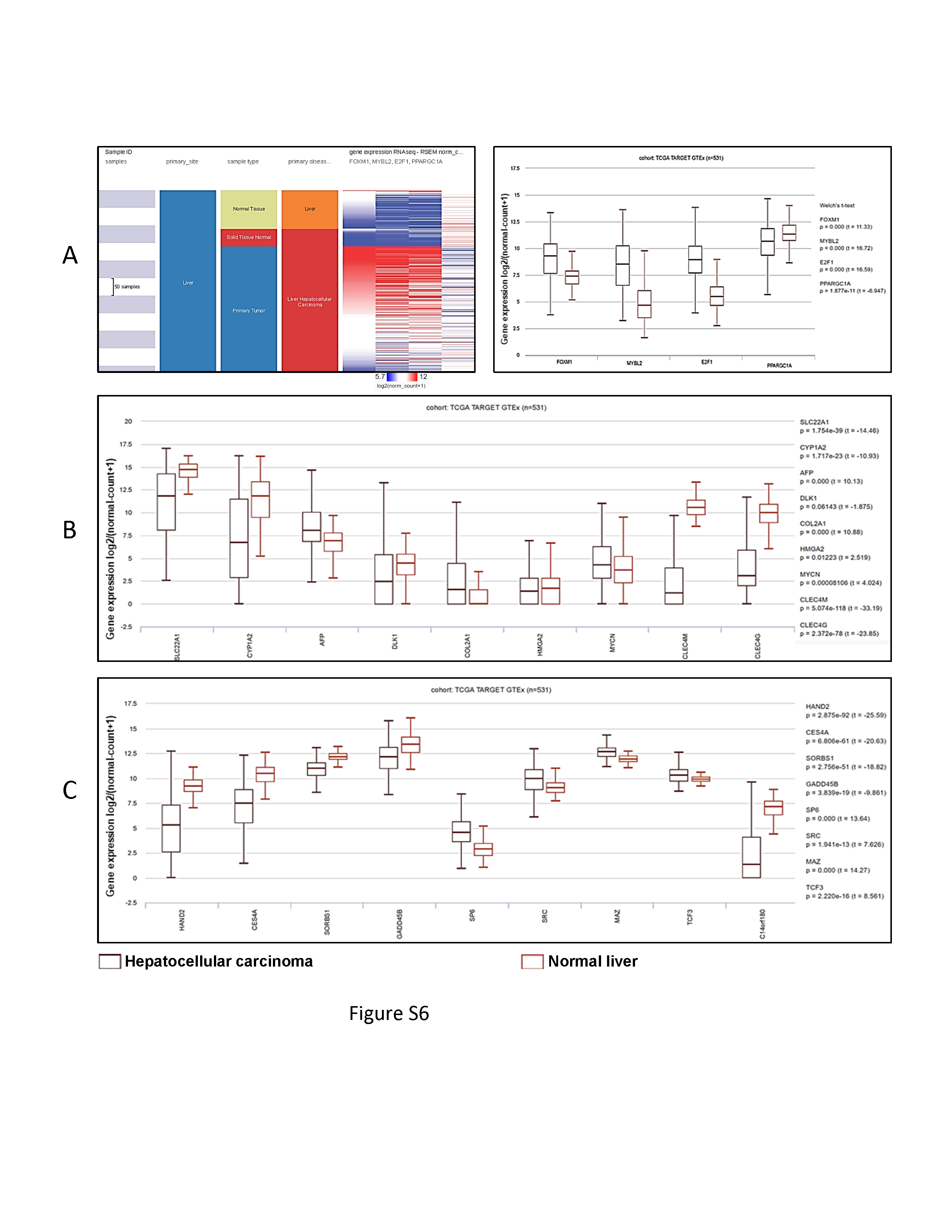

Supplement: Supplementary Figure S1 — Representative histological features of the 4 cases. (A) Tumor from case 1 was composed of large polygonal cells with round to oval hyperchromatic vesicular nuclei, small nucleoli, moderate amounts of eosinophilic cytoplasm, numerous mitoses and arranged mainly in acini and trabeculae. (B) Resection tumor from case 2 revealed sheets of medium sized polygonal cells with round hyperchromatic vesicular nuclei, inconspicuous nucleoli, focal cytoplasmic vacuolation and frequent mitoses. (C) Tumor section from case 3 demonstrated normal liver parenchyma (left upper) and adjacent tumor (right side), which was composed of small to medium sized atypical cells with vesicular nuclei, inconspicuous nucleoli, moderate amounts of clear to eosinophilic cytoplasm and increased mitoses. (D) Tumor from case 4 showed sheets of medium to large polygonal cells with large nuclei, prominent nucleoli and increased mitotic activity. (A–D) Hematoxylin-eosin stain; Original magnification: 200 x for all. [file DataSheet_1.zip › Image 6.TIF]

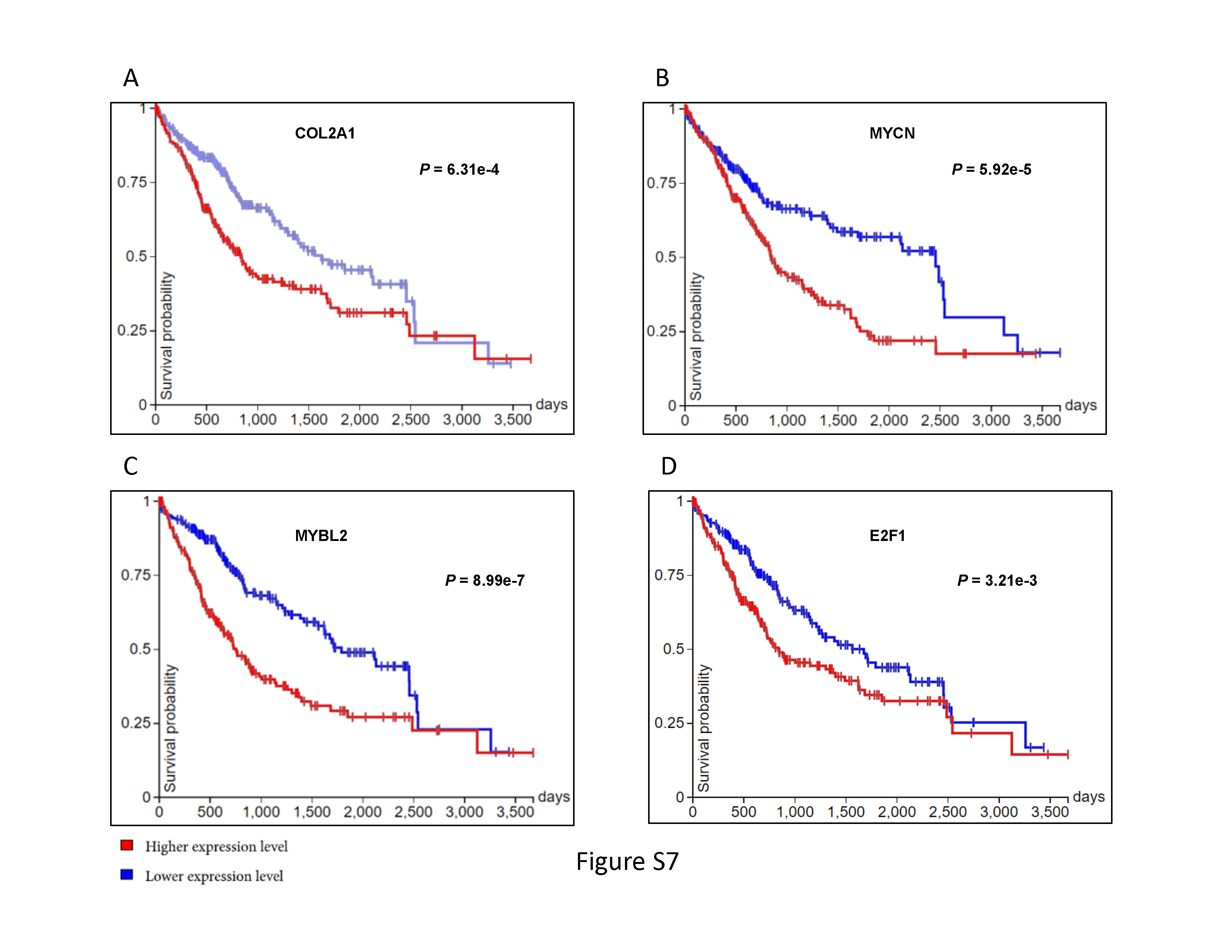

Supplement: Supplementary Figure S1 — Representative histological features of the 4 cases. (A) Tumor from case 1 was composed of large polygonal cells with round to oval hyperchromatic vesicular nuclei, small nucleoli, moderate amounts of eosinophilic cytoplasm, numerous mitoses and arranged mainly in acini and trabeculae. (B) Resection tumor from case 2 revealed sheets of medium sized polygonal cells with round hyperchromatic vesicular nuclei, inconspicuous nucleoli, focal cytoplasmic vacuolation and frequent mitoses. (C) Tumor section from case 3 demonstrated normal liver parenchyma (left upper) and adjacent tumor (right side), which was composed of small to medium sized atypical cells with vesicular nuclei, inconspicuous nucleoli, moderate amounts of clear to eosinophilic cytoplasm and increased mitoses. (D) Tumor from case 4 showed sheets of medium to large polygonal cells with large nuclei, prominent nucleoli and increased mitotic activity. (A–D) Hematoxylin-eosin stain; Original magnification: 200 x for all. [file DataSheet_1.zip › Image 7.TIF]
